# You Want it Sweeter: How Glycosylation Affects Plant Response to Oxidative Stress

**DOI:** 10.3389/fpls.2020.571399

**Published:** 2020-09-16

**Authors:** Marc Behr, Godfrey Neutelings, Mondher El Jaziri, Marie Baucher

**Affiliations:** ^1^Laboratoire de Biotechnologie Végétale, Université libre de Bruxelles, Gosselies, Belgium; ^2^UGSF—Unité de Glycobiologie Structurale et Fonctionnelle, UMR 8576, Université de Lille, CNRS, Lille, France

**Keywords:** abiotic stress, biotic stress, flavonoid, glucosidase, phytohormone, redox homeostasis, ROS—reactive oxygen species, UDP-glycosyltransferase (UGT)

## Abstract

Oxidative stress is a cellular threat which puts at risk the productivity of most of crops valorized by humankind in terms of food, feed, biomaterial, or bioenergy. It is therefore of crucial importance to understand the mechanisms by which plants mitigate the deleterious effects of oxidizing agents. Glycosylation of antioxidant molecules and phytohormones modifies their chemical properties as well as their cellular and histological repartition. This review emphasizes the mechanisms and the outcomes of this conjugation reaction on plant ability to face growing conditions favoring oxidative stress, in mirror with the activity of deglycosylating enzymes. Pioneer evidence bridging flavonoid, glycosylation, and redox homeostasis paved the way for numerous functional analyses of UDP-glycosyltransferases (UGTs), such as the identification of their substrates and their role to circumvent oxidative stress resulting from various environmental challenges. (De)glycosylation appears as a simple chemical reaction regulating the biosynthesis and/or the activity of a myriad of specialized metabolites partaking in response to pathogen and abiotic stresses. This outcome underlies the possibility to valorize UGTs potential to upgrade plant adaptation and fitness in a rising context of sub-optimal growing conditions subsequent to climate change.

## Introduction

Oxidative metabolism is a cornerstone of general cell biology. Plants are characterized by the ability to perform photosynthesis, a biological process supported by high rates of electron transfer in the thylakoids (Waszczak et al., 2018). The metabolism of energy factories, i.e., chloroplasts and mitochondria, generates reactive oxygen species (ROS, Vaahtera et al., 2014; Noctor et al., 2018). Cellular redox status is the balance between ROS abundance and the efficiency of the ROS scavenging system. Under optimal conditions, the deleterious effects of ROS are buffered by multiple ROS-scavenging mechanisms, resulting in a tightly adjusted redox homeostasis ensuring optimal cell metabolism. When ROS production overcomes the capacity of the scavenging systems, cells are facing a situation of oxidative stress. Plant cells have designed intricate signaling pathways to sense and react to this redox imbalance at multiple levels, a mechanism known as redox signaling, to restore redox homeostasis.

In addition to their intrinsic ROS production, plants cells are challenged by a large range of adverse conditions perturbing their redox homeostasis, such as heavy metals, pathogens, drought, salinity, high light, and extreme temperature (Schützendübel and Polle, 2002; Waszczak et al., 2018; Farooq et al., 2019). Oxidative stress is a cellular consequence of these conditions, as reviewed in (Bose et al., 2014; Pospíšil and Prasad, 2014; Anjum et al., 2015; Pospíšil and Yamamoto, 2017; Fryzova et al., 2018), which result in various developmental damages, such as growth arrest, leaf wilting, chlorosis, and impaired reproduction (Gray and Brady, 2016; Lv et al., 2019; Dubois and Inzé, 2020). At the physiological level, oxidative stress leads to lipid peroxidation, oxidative modifications of proteins and DNA damage (Pospíšil and Yamamoto, 2017; Huybrechts et al., 2019). Global warming is likely to increase the frequency and the severity of these events (Diffenbaugh et al., 2017), prompting to decipher and boost ROS scavenging as well as the dissipative mechanisms in species of economical and food interest (Gómez et al., 2019). To overcome their deleterious effects, ROS are scavenged through the actions of enzymes in charge of decreasing their oxidant capacity, such as superoxide dismutase (SOD) or catalase (CAT; Farooq et al., 2019) or by direct reaction with antioxidant molecules, such as redox-regenerable glutathione and ascorbate (Noctor et al., 2018), as well as classes of specialized metabolites such as flavonoids (Montoro et al., 2005; Agati et al., 2007; Nakabayashi et al., 2014). These canonical mechanisms are essentially conserved within the plant kingdom (He et al., 2018; Pan et al., 2020). ROS-removing systems also include molecular actors which indirectly affect redox homeostasis. For instance, some UGTs, which glycosylate a large range of metabolites, also partake in redox homeostasis. Together with glucosidases, UGTs rapidly shape the glycosylation status of a wide range of specialized metabolites to support plant response in various challenging environments. The precise regulation of the balance between glycosylation and deglycosylation applied to antioxidant molecules and to phytohormones allows plant to respond to environmental cues (Verma et al., 2016).

Glycosylation has emerged as a wide conjugation reaction of various molecules, as evidenced by the occurrence in plant extracts of multiple glycosylated forms of different classes of specialized metabolites. The variation in specialized metabolite profiles and the occurrence of UGTs among different species suggest that plant response to equivalent challenging conditions, e.g., redox imbalance, is species-dependent. This specificity highlights the plasticity of UGTs to specifically fit plant requirement, in terms of substrate affinity and environmental conditions (Gachon et al., 2005). This explains the great number of UGTs found in highly evolved plant species. The algae *Chlamydomonas reinhardtii* has only 1 protein with a UGT domain, *versus* 21 for the moss *Physcomitrella patens*, 115 for *Arabidopsis thaliana*, 168 for *Zea mays*, and 236 for *Populus trichocarpa* (Yonekura-Sakakibara and Hanada, 2011). Typically, these enzymes are able to use *in vitro* several substrates (Sun et al., 2019) leading therefore to the glycosylation of a myriad of metabolites with various biological functions *in planta*.

Consistent with the diversity of their substrates, UGTs are part of multiple biosynthetic and signaling networks partaking in redox homeostasis (Tiwari et al., 2016). First, UGTs are able to glycosylate specialized metabolites directly involved in ROS homeostasis, such as some flavonoids and terpenoids. Sugar substitution directly modifies the antioxidant potential when occurring in such substrates (Zheng et al., 2017), but also their cellular and tissular repartition (Taguchi et al., 2000) and retroactively regulates their corresponding biosynthetic pathway (Zhang and Liu, 2015). Second, a large range of phytohormones, which are widely reported to be important during plant developmental processes as well as during response to environmental stresses (Verma et al., 2016), undergoes glycosylation that modulate their activity.

Based on results reporting differential sensitivity of plants with altered *UGT* expression toward these stresses, we will explain and discuss how the glycosylation of selected substrates contribute to the equilibrium of the redox status during challenging conditions. In order to provide a reader’s guide, the main information dealing with the UGTs which have been thoroughly investigated so far are presented in **Table 1**. All these results are depicted by the UGT potential substrate(s) and will be further explained in the upcoming sections.

**Table 1:** Main UGTs described in this review.

UGT	Substrate	Role *in planta*	Inducing factor
**Flavonoids**			
**UGT71C1 (Arabidopsis)**	Flavonoids, lignans	Flavonoid aglycones are potentially more antioxidant than their glycosylated forms, explaining the better tolerance of knockout mutants to methyl viologen.	Unknown
**UGT73B1, UGT73B2, UGT73B3 (Arabidopsis)**	Flavonoids		Pathogen infection, SA, methyl jasmonate, oxidative stress
**UGT78A14** **(*Camellia sinensis*)**	Kaempferol, quercetin	Kaempferol and quercetin glycosylation products have higher ROS scavenging activity (FRAP, DPPH and ABTS) than their corresponding aglycones and contribute to cold stress tolerance.	Cold stress
**UGT79B2/UGT79B3 (Arabidopsis)**	Cyanidin/cyanidin-3-*O*-glucoside	Glycosylation of anthocyanins results in their storage in the vacuole, derepressing the product feedback inhibition on PAL and increasing total anthocyanin content. Plants are therefore more tolerant to cold, salt, and drought stresses.	Cold, salt, and drought stresses
**UFGT2** **(*Zea mays*)**	Quercetin, kaempferol	Flavonoids can protect the plant from oxidative stress resulting from exposure to salt, H_2_O_2_ and high osmotic condition. Glycosylation supports the biosynthesis of these flavonoids.	Salt, drought and oxidative stresses
**Phytohormones**			
**SDG8i** **(*Sporobolus stapfianus*)**	Strigolactones	Overexpression of this gene in Arabidopsis results in higher tolerance to salt, freezing and drought stresses. Strigolactones favour the biosynthesis of anthocyanins and strigolactones glycosylation may ease their translocation between different organs.	Water stress
**UGT71B6, UGT71B7, UGT71B8 (Arabidopsis)**	ABA	These UGTs maintain an optimal ABA content under non-limiting growing conditions and may mitigate ABA response during salt and osmotic stress.	ABA, salt and osmotic stresses
**UGT74E2 (Arabidopsis)**	Indole-3-butyric acid	The enzymatic activity of UGT74E2 may modify auxin gradient and distribution to support plant acclimation under drought and salt stress conditions, in interaction with flavonoids.	Osmotic, oxidative, ultraviolet B and salt stresses
**UGT90A1** **(*Oryza sativa*)**	Possibly auxins and cytokinins	Overexpression in rice and Arabidopsis leads to higher tolerance to cold and salt stress, with lower accumulation of ROS and higher catalase and soluble PRX activities.	Low temperature, salt stress
**Miscellaneous**			
**TOGT** **(*Nicotiana tabacum*)**	Scopoletin	Scopoletin is an antiviral and a potent antioxidant in plant defense response. Scopoletin regulates ROI accumulation in cells surrounding necrosis (as substrate of PRX or direct ROI scavenger).	SA- and pathogen-inducible
**UGT73B3, UGT73B5 (Arabidopsis)**	Phytoprostanes, camalexin degradation products	These UGTs contribute to the regulation of redox status and general detoxification of ROS-reactive specialized metabolites, limitation of cell death establishment during HR.	Early-SA induced gene, paraquat, ozone, bacteria
**UGT85A5** **(Arabidopsis)**	Unknown	Ectopic expression increases tolerance to salt stress, as indicated by lower loss of chlorophyll and lower malondialdehyde equivalents content.	Salt stress
**UGT91Q2** **(*Camellia sinensis*)**	Nerolidol	Nerolidol glucoside has a higher ROS scavenging activity than its aglycone, possibly protects PSII during cold stress and prevents lipid peroxidation.	Cold stress

ABA, abscisic acid; ABTS, 2,2’-azino-bis(3-ethylbenzothiazoline-6-sulfonic acid); DPPH, 2,2-diphenyl-1-picrylhydrazyl; FRAP, ferric reducing ability of plasma; HR, hypersensitive response; PAL, phenylalanine ammonia lyase; PRX, peroxidase; PSII, photosystem II; ROI, reactive oxygen intermediates; ROS, reactive oxygen species; SA, salicylic acid.

Additional details and corresponding references are provided in the main text.

We will first sum up the first elements highlighting the involvement of some UGTs toward chemically-induced oxidative stress. The second part will be devoted to the study of UGTs related to redox homeostasis during pathogen infection. The third part will explain how the glycosylation of antioxidant molecules can mitigate oxidative stress arising from abiotic stresses. Finally, the last part will address the participation of the glycosylation of selected phytohormones during plant response to abiotic constrains. The conclusion will open perspectives to the potential interactions between glycosylation and redox retrograde signaling.

## First Evidences Connecting Glycosylation to Plant Response to Oxidative Stress

The first molecular characterization of an UGT, demonstrated to guide flavonoid glycosylation, was reported in maize (Dooner and Nelson, 1977). The sequencing and the annotation of several plant genomes, especially of model species such as *A. thaliana*, highlighted the diversity of the UGT families. Since then, an unceasing effort has been undertaken to decipher the cellular functions of UGTs and to provide evidences for their roles *in planta*. Meanwhile, the importance of flavonoids regarding protection to oxidative damages was highlighted (Landry et al., 1995), underlying the relevance of studying flavonoid glycosylation in the context of oxidative stress. One of these first studies focused on the role of Arabidopsis *UGT73B1*, *UGT73B2*, and *UGT73B3* concerning methyl viologen (MV) tolerance (Chae et al., 2006). In the acceptor side of photosystem I, MV catalyzes reduction of oxygen into superoxide ion $O_{2}^{•-}$, a potent oxidant agent (Noctor et al., 2016). MV is widely used experimentally to specifically induce ROS production. While Arabidopsis T-DNA insertional mutants of each of these genes did not display any obvious phenotype under normal growth conditions, they turned to be more tolerant to MV in terms of biomass and chlorophyll content as compared to the wild-type (25- to 55-fold higher chlorophyll a content at 1.5 µM MV and higher total chlorophyll content). Compared to the WT, this increased tolerance to MV may have two origins. The first one is a lower $O_{2}^{•-}$production, as suggested by the decreased nitroblue tetrazolium (NBT) staining of the *ugt73b2* mutant following MV exposure (Kim et al., 2010). The second one is an increased redox buffer potential able to maintain a ROS concentration compatible with survival, which lays in more abundant/efficient ROS scavenging through enzymatic, such as SOD and CAT, and/or non-enzymatic (antioxidant molecules) systems. The tolerance to MV in *ugt73b1*, *ugt73b2*, and *ugt73b3* mutants might be a consequence of the accumulation of flavonoid aglycones (Lim et al., 2008; Kim et al., 2010), as proteins of these UGT families glycosylate flavonoids (Jones et al., 2003; Chae et al., 2006; Lim et al., 2008; Sun et al., 2019). For instance, UGT73B1 and UGT73B2 show *in vitro* glucose-conjugating activity toward the flavanones eriodictyol and naringenin, the flavones apigenin and luteolin, and the flavonols kaempferol and quercetin (Kim et al., 2006b; Kim et al., 2006a). As kaempferol and quercetin glycosides naturally occur in Arabidopsis leaves and flowers (Jones et al., 2003), it is likely that they are natural substrates of these UGT73. Since quercetin and kaempferol are major flavonoids (Deng and Lu, 2017), we may hypothesize that the depletion of their glycosylated forms entails increased pool of their aglycones, explaining the tolerance to MV-induced oxidative stress in the *ugt73b* mutants. In addition, exogenous naringenin was shown to protect chloroplasts from oxidative damages through activation of H_2_O_2_ scavenging mechanisms (Yildiztugay et al., 2020), which may also account for increased MV tolerance of *ugt73b1* and *ugt73b2* mutants.

Results similar to those reported for *ugt73b2* mutant have been obtained with an *ugt71c1* mutant. While no obvious phenotype was observed in control conditions, this line retains higher total chlorophyll and carotenoid content following MV treatment (0.5 and 1 µM) as compared to the wild-type (Lim et al., 2008). When facing a MV concentration of 1.5 µM, the radical scavenging activity of seedlings, as assessed with 2,2-diphenyl-1-picrylhydrazyl (DPPH) assay, was significantly higher in the *ugt71c1* mutant than in the wild-type. The expression of eight genes involved in ROS response (such as *ALTERNATIVE OXIDASE*, *CAT*s, *SOD*s, and *PEROXIREDOXIN*) was lower in the *ugt71c1* mutant under various MV concentrations, as compared to the wild-type (Lim et al., 2008). In absence of MV, the expression of these genes was similar in the two genotypes. This strongly suggests that MV tolerance of this line does not rely on ROS scavenging by enzymatic detoxication process. As further explained below, a possible involvement of flavonoids is suspected.

In point of fact, the *ugt71c1* mutant showed decreased amounts of quercetin 3,7-*O*-glucoside and kaempferol 3,7-*O*-glucoside (25% and 70% of wild-type level, respectively; Lim et al., 2008) as well as reduced lariciresinol- and pinoresinol-glucosides content (Okazawa et al., 2014). In addition to quercetin and luteolin (Lim et al., 2003), recombinant UGT71C1 is able to glycosylate the lignans lariciresinol and pinoresinol (Okazawa et al., 2014). Lariciresinol efficiently inhibits lipid peroxidation *in vitro* (Zhang et al., 2004). Isolariciresinol is an isomer of lariciresinol and its 4’-β-D-glucoside form is less antioxidant than its aglycon (Baderschneider and Winterhalter, 2001). These data are consistent with increased MV tolerance of the *ugt71c1* mutant. Lignans are biosynthesized in response to various adverse conditions, comprising oxidative stress (Paniagua et al., 2017). Interestingly, reduced neolignans such as isodihydrodehydrodiconiferyl alcohol contribute to cytosolic H_2_O_2_ scavenging in poplar during xylem differentiation (Niculaes et al., 2014). This further suggests that lignan glycosylation may impact on redox homeostasis.

**Figure 1:**
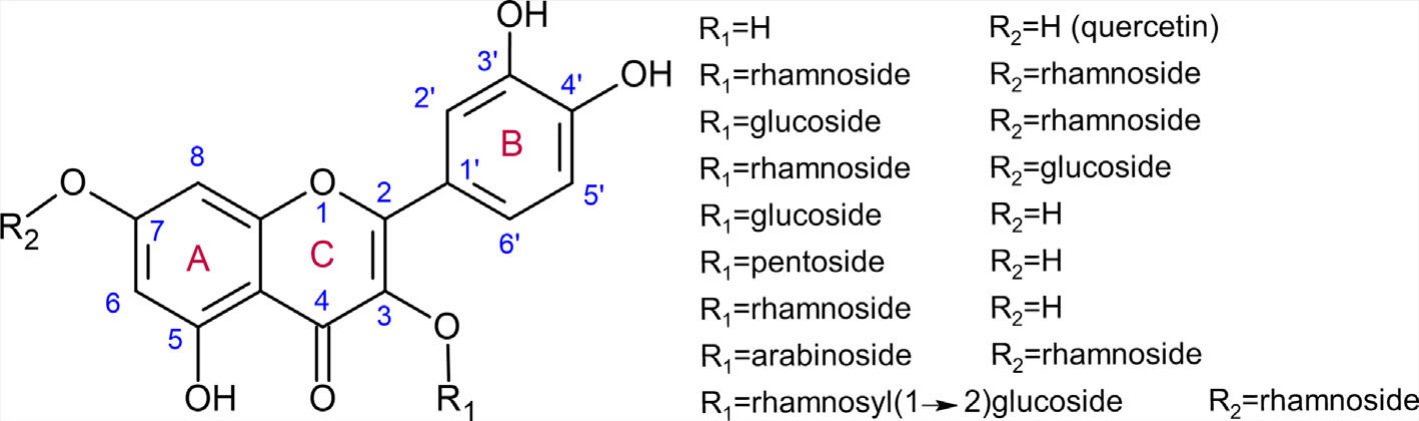
Quercetin and examples of quercetin glycosides detected in various organs of *Arabidopsis thaliana* using ultraperformance liquid chromatography–photodiode array–electrospray ionization/quadrupole time-of-flight/mass spectrometry (Yonekura-Sakakibara et al., 2008).

**Figure 2:**
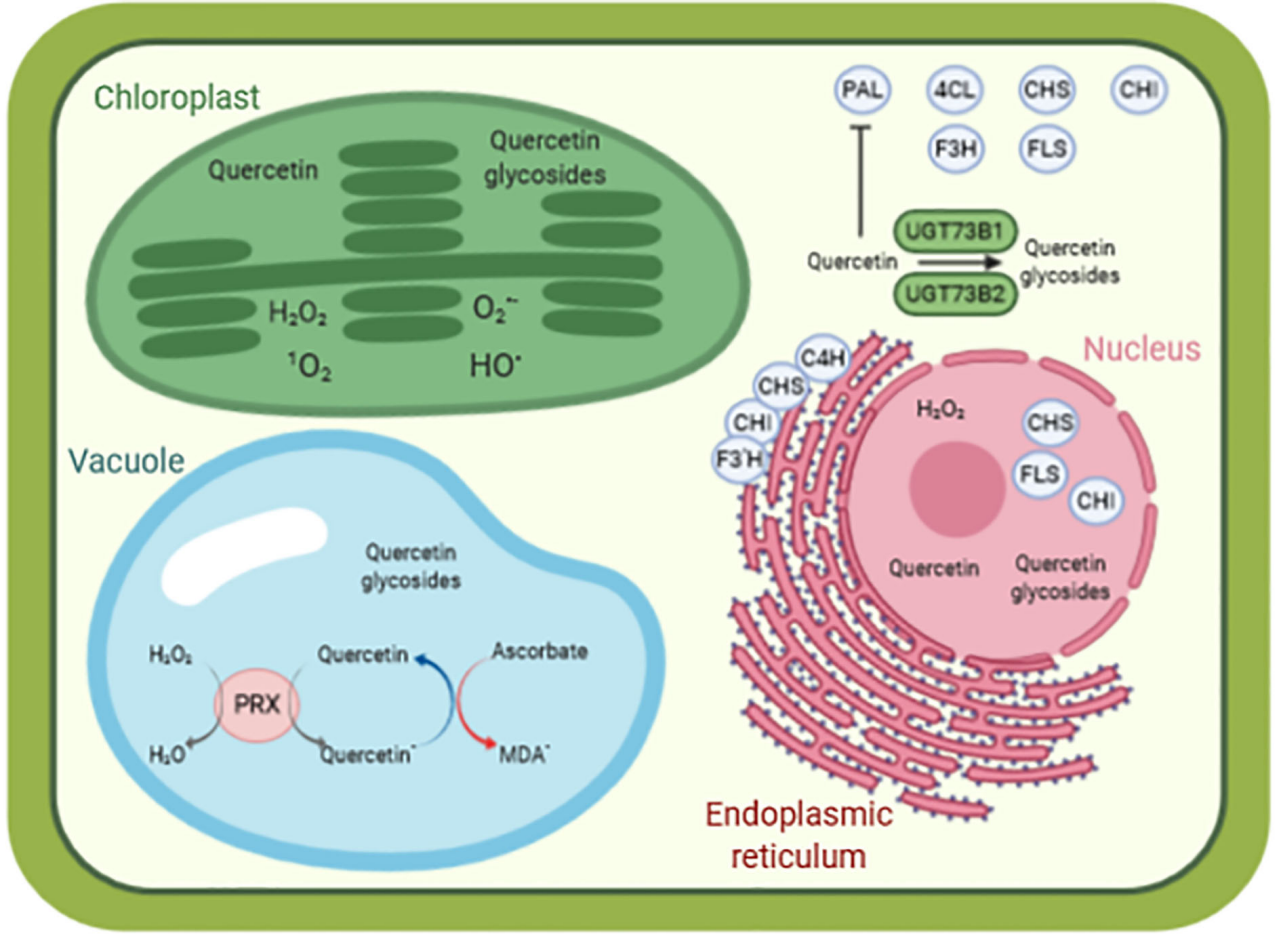
A simplified model of quercetin as antioxidant molecule in a cellular context. The biosynthesis of quercetin is performed by enzymes located in the cytosol (PAL, C4H, CHS, F3H, FLS), anchored to the endoplasmic reticulum (C4H, CHS, CHI, F3’H) and present in the nucleus (CHS, CHI, FLS). Quercetin is glycosylated by various UGTs, such as UGT73B1 and UGT73B2 in *Arabidopsis thaliana*. Quercetin and quercetin glycosides are found in various compartments (such as cytosol, nucleus, chloroplasts, and vacuole), contributing to redox homeostasis and preventing oxidative damages caused by reactive species, for instance, in the chloroplasts. In the vacuole, quercetin reduces H_2_O_2_ to H_2_O in a peroxidase-dependent reaction, leading to the formation of quercetin radical. This radical is then recycled back to quercetin by oxidation of ascorbate to monodehydroascorbate radical. Data from (Burbulis and Winkel-Shirley, 1999; Saslowsky et al., 2005; Fujino et al., 2018; Khorobrykh et al., 2020). ^1^O_2_ singlet state of molecular oxygen, HO^•^ hydroxyl radical, H_2_O_2_ hydrogen peroxide, and O2^•−^ superoxide anion radical. 4CL, 4-coumaroyl:CoA-ligase; C4H, cinnamate 4-hydroxylase; CHI, chalcone isomerase; CHS, chalcone synthase; F3H, flavanone 3-hydroxylase; F3’H, flavonoid 3’-hydroxylase; FLS, flavonol synthase; MDA^•^, monodehydroascorbate radical; PAL, phenylalanine ammonia lyase; PRX, peroxidase. Picture designed with Biorender.

Flavonoid aglycones are considered as more effective antioxidants than their glycosides (Rice-Evans et al., 1996; Hopia and Heinonen, 1999; Baderschneider and Winterhalter, 2001; Montoro et al., 2005). Zheng and colleagues showed that hydroxyl groups in B-ring and C-ring (**Figure 1**) contribute mainly to the antioxidative activities of quercetin and its glucosides, as compared with A-ring (Zheng et al., 2017). Rice-Evans et al. (1996) also demonstrated in quercetin that i) blocking the 3-hydroxyl group in the C-ring with a glycoside and ii) removing the 3-hydroxyl group in the C-ring, reduces its antioxidant activity. Some examples of quercetin derivatives detected in Arabidopsis are depicted in **Figure 1**. Dihydroxy B-ring substituted flavonoids such as quercetin and luteolin occur in the vacuole as well as in the chloroplastic and nuclear compartments, where they scavenge ROS with different mechanisms (Agati et al., 2012; Chapman et al., 2019). In the vacuole, they reduce H_2_O_2_ to H_2_O in a peroxidase-dependent reaction (mechanism extensively explained in Pourcel et al., 2007), leading to the formation of flavonoid radicals (**Figure 2**). Ascorbate recycles these radicals to their reduced form, allowing the reduction of additional H_2_O_2_ molecules (Yamasaki et al., 1997; Pérez et al., 2002; Ferreres et al., 2011). The main vacuolar peroxidase from *Catharanthus roseus* showed higher affinity for quercetin than for quercetin-3-*O*-arabinoside (Km of 0.045 mM for quercetin vs. 1.589 mM for the glycoside), providing a biological explanation to the antioxidant value of flavonoid aglycones and their high H_2_O_2_ scavenging capacity (Ferreres et al., 2011). When localized in chloroplasts, these flavonoids efficiently scavenge singlet oxygen, while in the nucleus, they protect DNA from oxidative damages (**Figure 2**) (Naoumkina and Dixon, 2008; Brunetti et al., 2019). The main structural feature explaining the free radical scavenging capacity of flavonoids consists in the high reactivity of their hydroxyl substituents, as explained by (Heim et al., 2002):

$$Flavonoid-OH+R^{\cdot }\to Flavonoid-O^{\cdot }+RH$$

The results obtained in Arabidopsis lines with altered *UGT71* and *UGT73* expression demonstrate that glycosylation directly modifies plant cellular redox scavenging potential and demonstrate the role of UGTs in redox homeostasis.

## Glycosylation Regulates Redox Status During Response to Pathogens

UGT73B3 and UGT73B5 were also investigated in the context of plant response to bacterial infection. An untargeted metabolite analysis show that three kaempferol glucosides are slightly more accumulated in the wild-type than in the *ugt73b3* and *ugt73b5* mutants (Simon et al., 2014), providing additional evidence that kaempferol is a substrate of UGT73B3 and UGT73B5 (Kim et al., 2006b; Kim et al., 2006a). The *ugt73b3 ugt73b5* double mutant was also characterized by a lower ascorbate pool (Simon et al., 2014), which is part of the H_2_O_2_ scavenging system in combination with ascorbate peroxidase (Asada, 1992; Smirnoff and Arnaud, 2019). During hypersensitive response (HR) to *Pst*-*AvrRpm1*, an avirulent strain of the biotrophic bacteria *Pseudomonas syringae* pv. *tomato*, *ugt73b3 ugt73b5* double mutant exhibited, as compared to wild-type, slightly enhanced cell death (ca. 10%) as measured by electrolyte leakage in the leaves (Simon et al., 2014). While no genotypic difference in H_2_O_2_ content was observed in absence of infection, the interaction between *Pst*-*AvrRpm1* and the single or double mutants resulted, as compared to wild type, in a higher accumulation of H_2_O_2_ (18%, 36%, and 42% in *ugt73b3*, *ugt73b5*, and *ugt73b3 ugt73b5*, respectively), which usually activate defense mechanisms (Smirnoff and Arnaud, 2019). Since this H_2_O_2_ accumulation did not correlate with decreased bacterial growth in leaves (Simon et al., 2014), it seems that the higher sensitivity of the double mutant is directly related to the absence of UGT73B3 and UGT73B5 and not to subsequent downstream consequences. This also suggests the important role of the ROS scavenging system, including UGT73B3 and UGT73B5, in response to *Pst*-*AvrRpm1* (Simon et al., 2014). It is also surprising that, while kaempferol prevents $O_{2}^{•-}$ accumulation caused by MV in the *ugt73b1-2-3* backgrounds (Chae et al., 2006), it does not seem efficient to prevent H_2_O_2_ accumulation generated by *Pst*-*AvrRpm1* infection during HR in *ugt73b3* and *ugt73b5* mutants. In other words, the production of ROS during HR overcomes the redox scavenging capacity of the cell. However, this ROS burst, whose role is to enable a more efficient defense system, is not able to lower bacterial growth in infected leaves. This may be hypothetically related to the different metabolic profile of *ugt73b3* and *ugt73b5* after *Pst*-*AvrRpm1* infection, explaining the large spreading of the pathogen in the mutants. For instance, both glutathione content and oxidation status were altered in the mutants as compared to wild-type. *UGT73B3* and *UGT73B5* are co-expressed with genes associated to cellular redox status and detoxification (such as several *glutathione S-transferases* and ROS-induced genes). In the wild-type, oxidative stress, here caused by a pathogen attack, induces the expression of this subset of genes, which in turn contributes to limit cell death establishment during HR through regulation of redox homeostasis. It is important to notice that the Arabidopsis *tt4* mutant, depleted in flavonoids and kaempferol glycosides, displays essentially the same sensitivity to *P*. *syringae* than its corresponding wild-type (Hagemeier et al., 2001), indicating that these molecules do not significantly account for the higher sensitivity of the *ugt73b3* and *ugt73b5* mutants. Instead, several elements suggest that during HR, UGT73B3 and UGT73B5 may accept as substrates, i) phytoprostanes, which are reactive lipids accumulating under oxidative conditions, to change their redox activity, and ii) phytoalexin by-products, such as hydroxycamalexin, whose accumulation is induced by H_2_O_2_ and several microorganisms (Simon et al., 2014). Such hypotheses remain however to be further validated.

Two tobacco salicylic acid- and pathogen-inducible UGTs (TOGTs) belonging to the UGT73B subfamily preferentially catalyze the glycosylation of the hydroxycoumarin scopoletin into scopolin (Fraissinet-Tachet et al., 1998; Chong et al., 2002). Simultaneous down-regulation of the two corresponding genes with an antisense construction coupled to tobacco mosaic virus (TMV) infection decreased scopoletin and scopolin content (three- to four-fold), enhanced lesion size (23 to 28%) while increasing reactive oxygen intermediates (ROI, i.e., $O_{2}^{•-}$and H_2_O_2_) content (1.5- to 2.2-fold) in comparison with empty-vector transformed plants. The diminution of the scopoletin-scopolin pool therefore leads to increased accumulation of ROI. Besides, treatment of tobacco protoplasts with scopoletin significantly reduces TMV multiplication in a dose-dependent manner (Chong et al., 2002). Overall, scopoletin may act at two distinct levels: as an antiviral molecule in the tissues infected by TMV and as an antioxidant in cells surrounding the infected area to mitigate the effects of HR-induced ROI accumulation. The scopoletin antioxidant capacity is probably related to its ability to directly scavenge ROI and/or to its use as a substrate for peroxidase-mediated H_2_O_2_ detoxification (Yamasaki et al., 1997; Chong et al., 2002; Agati et al., 2012).

Overexpression of *TOGT* in tobacco results in both scopoletin and scopolin over-accumulation as compared to wild-type (Gachon et al., 2004), demonstrating that up-regulation of glycosylating activity toward a specific substrate does not necessarily result in lower accumulation of the corresponding aglycone form. This may be explained by their differential accumulation within cellular compartments (Taguchi et al., 2000). In tobacco T-13 cells, scopolin indeed preferentially accumulates in the vacuole (93% of the total pool), while the proportion of scopoletin is higher in the protoplast excluding the vacuole (70% of the total pool) (Taguchi et al., 2000). Glycosylation therefore decreases scopoletin cytoplasmic content by increasing vacuolar scopolin content, and this conversion mainly occurs in the cytosol (Taguchi et al., 2000). The transcription and activity of phenylalanine ammonia lyase (PAL), the first enzyme of the phenylpropanoid biosynthesis pathway from which is derived scopoletin, is well known to be negatively regulated by downstream metabolic products, such as *trans*-cinnamate and flavonols (**Figure 2**) (Zhang and Liu, 2015). Therefore, glycosylation and subsequent accumulation of its glycosylated form in the vacuole favors scopoletin biosynthesis and associated antioxidant activity by avoiding PAL feedback inhibition.

Thus, UGT activity may have, for various substrates, an important role in the homeostasis of their respective metabolic pathway. Such a regulation is of crucial importance for maintenance of redox stability when the aglycone/glycosylated form of a given molecule is involved in this subtle equilibrium. Collectively, these results show that UGT may participate in redox homeostasis through a panel of biochemical mechanisms in order to support global plant response to oxidative stress related to pathogen infection. Scopoletin accumulation supported by TOGT activity both inhibits viral multiplication and buffers the redox status of infected cells, while UGT73B3 and UGT73B5 may detoxify molecules produced by infected cells.

## Glycosylation of Specialized Metabolites Shapes Oxidative Status During Abiotic Stresses

Abiotic stresses result in accumulation of ROS and disturbance of the redox homeostasis (Keunen et al., 2013; Xie et al., 2019). Plants exposed to these stresses usually accumulate more antioxidant molecules, such as phenolics, carotenoids, and tocopherols (Wang and Frei, 2011) to re-equilibrate their oxidative balance. Consistent with the accumulation of phenolics, PAL is strongly induced at both transcriptional and enzymatic activity levels (Wang and Frei, 2011; Xing et al., 2013), similarly to other genes involved in flavonoids biosynthesis (Cheynier et al., 2013). Flavonoids accumulation significantly increases tolerance to oxidative stress resulting from high irradiance or drought in various plant species (Agati et al., 2007; Gómez et al., 2019).

Strikingly, there is also a pure energy rationale behind specialized metabolites biosynthesis (Selmar and Kleinwächter, 2013). Various abiotic stresses, such as drought and high temperature, result in partial stomatal closure, implying decreased requirement of ATP and reduction equivalents (NADPH+H^+^) for CO_2_ fixation in the Calvin cycle. Consequently, the pool of NADP^+^ accepting electrons becomes depleted. Instead, these electrons would be transferred to oxygen, resulting in the production of superoxide radicals. The biosynthesis of highly reduced specialized metabolites (such as monoterpenes, alkaloids, aromatic amino acids, and phenolics) aims at regenerating the pool of NADP^+^ through consumption of the reduction equivalents which cannot be directed to the Calvin cycle (Selmar and Kleinwächter, 2013).

The expression of several *UGT*s is induced during particular abiotic conditions (Rehman et al., 2018). Moreover, *UGT*s may also be co-expressed with genes from a specific biosynthesis pathway such as flavonols and anthocyanins (Tiwari et al., 2016). These observations suggest that UGTs partake in plant global response to abiotic stresses. For instance, the down-regulation of two *UGT*s from tea, *UGT78A14* and *UGT91Q2*, was shown to result in lower tolerance to cold stress (Zhao et al., 2019; Zhao et al., 2020), which also triggers oxidative stress. Interestingly, expression of these two genes is cold-inducible, suggesting that their function *in planta* is closely related to tolerance to this abiotic stress. UGT78A14 accepts kaempferol and quercetin as substrate, with a higher affinity for the first. *UGT78A14* downregulated tea plants show i) decreased accumulation of total flavonoids, kaempferol-3-*O*-glucoside, kaempferol-7-*O*-glucoside, and kaempferol diglucoside (possibly *via* feedback gene inhibition), ii) reduced ROS scavenging capacity as measured by ferric reducing ability of plasma (FRAP) and DPPH assays, and iii) impaired tolerance to cold stress (lower optimal quantum yield Fv/Fm, an indicator of photoinhibition, after exposure to cold stress). Their leaves are characterized by an increased accumulation of superoxide radicals and H_2_O_2_, which may result in lipid peroxidation and decreased photosynthetic efficiency. The UGT78A14 glycosylation products of kaempferol and quercetin have higher ROS scavenging activity than their corresponding aglycones as assessed by FRAP, DPPH and 2,2’-azino-bis(3-ethylbenzothiazoline-6-sulfonic acid) (ABTS) assays (Zhao et al., 2019). This result is contradictory to previous observations, which stated that flavonoid aglycones show higher antioxidant capacity than their glycosylated forms, as demonstrated for quercetin and luteolin using another method, the Trolox equivalent antioxidant activity (TEAC) test (Rice-Evans et al., 1996; Hopia and Heinonen, 1999; Montoro et al., 2005). Furthermore, increased kaempferol and quercetin content obtained by transient overexpression in tobacco of the *Crocus sativus CsBGLU12*, coding for a β-glucosidase active on flavonol glycosides, resulted in leaves more tolerant to ultraviolet B, dehydration and salt exposure, as shown by higher chlorophyll content and lower lipid peroxidation after stress application (Baba et al., 2017). We may suggest that the phenotypes related to oxidative stress tolerance observed in tea with down-regulation of *UGT78A14* (Zhao et al., 2019) and tobacco with over-expression of *CsBGLU12* (Baba et al., 2017) are partially explained by different pools of antioxidant molecules, which are not restricted to the few flavonoids developed in this review. Other specialized metabolites, whose molecular structures and contents are highly variable between distinct species, may explain this difference. Moreover, Zhao and colleagues (2019) investigated tolerance to cold stress, while Baba and colleagues (2017) focused on ultraviolet B, dehydration and salt exposure. While all these conditions result in oxidative stress, they most likely do not invoke the same cellular and molecular consequences and responses. For instance, flavonoids involved in protection against ultraviolet B may be preferentially accumulated in the cell wall to protect the cell. In order to cross the plasma membrane, these molecules should be slightly apolar and therefore not glycosylated. Consequently, the glycosylation pattern of a given molecule may not uniformly protect the plant against a range of various stresses. This discrepancy may also lie in the different substitution patterns of investigated flavonoids. Indeed, the glucose substitution pattern of a given aglycone is important to explain its antioxidant potential, as demonstrated for quercetin (Zheng et al., 2017) and previously explained. Two UGT78A14 alleles are reported to yield kaempferol-3-*O*-glucoside, kaempferol-7-*O*-glucoside, and kaempferol diglucoside in different proportions (Zhao et al., 2019), while UGT73B2 preferentially glucosylates kaempferol in the 3-hydroxyl position (Kim et al., 2006a). These different substitution patterns were reported to result in differential antioxidant properties for quercetin and related glycosylation or methoxylation derivatives (Rice-Evans et al., 1996; Montoro et al., 2005). In Arabidopsis, 32 flavonol glycosides showing variable antioxidant capacities have been identified (**Figure 1**) (Yonekura-Sakakibara et al., 2008; Chapman et al., 2019). Reporting of differential ROS scavenging activity between related molecules should therefore be carefully described, for instance, in terms of hydroxyl group substitution, and interpreted with caution.

The maize *UDP glucose:flavonoid glucosyltransferase 2* (*UFGT2*) supports plant response to various abiotic stresses and rescues the flavonols deficiency in the Arabidopsis *ugt78d2* mutant (Li et al., 2018). Arabidopsis *UFGT2* overexpressing lines displayed lower accumulation of H_2_O_2_ when facing oxidative stress following salt, osmotic, or H_2_O_2_ conditions while two knock-out maize mutants showed the opposite trend (Li et al., 2018). Quantification of flavonols in these different lines coupled to *in vitro* enzymatic activity consistently demonstrate that UFGT2 glycosylates quercetin and kaempferol. Similarly to *TOGT* (Gachon et al., 2004), overexpression of *UFGT2* in Arabidopsis not only increases the glycosylated forms of these two flavonols, but also the accumulation of their aglycones, through higher expression of core flavonol biosynthesis genes. Consistent with the previously described scopoletin and scopolin translocation mechanism (Taguchi et al., 2000), this equilibrium depends on the differential cellular compartmentalization of these molecules (**Figure 2**). The transport from the site of flavonoids biosynthesis, i.e., the endoplasmic reticulum (ER), to the tonoplast is highly favored by conjugation with either a sugar moiety (resulting in a glycosylated molecule) or a glutathione *S*-transferase group (**Figure 2**). These conjugations therefore maintain flavonoid homeostasis in the cytosol and support their corresponding biosynthetic pathways (Agati et al., 2012).

Anthocyanins are potent antioxidant molecules (Gould, 2004) and they are biosynthesized in response to several stresses. For instance, MV-induced accumulation of chloroplastic H_2_O_2_ enhances the expression of genes of the anthocyanin biosynthesis pathway as well as anthocyanin content in various Arabidopsis genetic backgrounds (Maruta et al., 2014). The silencing of a major chloroplastic ROS scavenging gene, coding for an ascorbate peroxidase, also resulted in enhanced anthocyanin content (Maruta et al., 2014). Flavonoids present in the chloroplasts are known to scavenge the highly reactive singlet oxygen (^1^O_2_) in order to protect the function of this organelle vs. oxidative stress (Agati et al., 2007). This result has been confirmed using Arabidopsis genotypes overaccumulating flavonoids, including overexpressors of *MYB12* and *PAP1* regulating flavonols and anthocyanins biosynthesis, resulting in better tolerance to MV and drought stress (Nakabayashi et al., 2014). Similarly to flavonols, anthocyanins undergo glycosylation and this conjugation has deep consequences under cold, salt, and drought stresses. For instance, overexpression of *UGT79B2* and *UGT79B3*, which glycosylate cyanidin and cyanidin 3-*O*-glucoside using UDP-rhamnose as sugar donor, resulted in increased tolerance when facing these three stresses, while RNAi line and CRISPR mutant showed enhanced sensitivity (Li et al., 2017). These phenotypes are most likely explained by the PAL feedback repression of anthocyanin intermediates, as previously noted for scopoletin and kaempferol. Indeed, anthocyanins preferentially locate in the vacuole, while their biosynthesis takes place in the smooth ER (Landi et al., 2015). In addition, two anthocyanins were detected in hydro-alcoholic extracts of vacuoles isolated from protoplasts of *C*. *roseus* (Ferreres et al., 2011). Once again, these two genes were strongly induced in response to cold, salt, and drought treatments. These genes are part of the cold-regulated genes subset, whose expression may be regulated by the transcription factor CBF1 to scavenge ROS under cold conditions. DAB and NBT staining, together with antioxidant FRAP assay, highlighted the correlation between expression of *UGT79B2* and *UGT79B3* and ROS scavenging capacity, demonstrating the importance of anthocyanin glycosylation in redox homeostasis under several adverse conditions (Li et al., 2017).

The cold-inducible *OsUGT90A1* was isolated from *Oryza sativa* based on its identification as most probable candidate gene explaining the *LOW TEMPERATURE SEEDLING SURVIVABILITY* QTL (Shi et al., 2020). As shown by these authors, *OsUGT90A1* overexpressing rice and Arabidopsis lines display lower electrolyte leakage following exposition at 10°C, 4°C, and −2°C. Membrane integrity was more preserved in these lines because of lower accumulation of ROS, as detected by NBT staining, in addition to higher CAT and soluble peroxidase activities after cold exposure. Opposite results were obtained in two lines with strongly decreased *OsUGT90A1* expression. This gene also increases salt tolerance. While the corresponding enzyme is annotated to glycosylate anthocyanins, no striking differences between these lines were observed in the flavonoid and anthocyanin content. The authors rather suggest, based on modified plant phenotype (longer shoots and shorter roots in line overexpressing *OsUGT90A1*), that OsUGT90A1 may accept auxins and cytokinins as substrates, but no enzymatic activity assays have been performed to further confirm this hypothesis.

Down-regulation of tea *UGT91Q2* reduces nerolidol (a volatile sesquiterpene alcohol) and nerolidol glucoside content, ROS scavenging activity (DPPH assay) and cold stress tolerance (Zhao et al., 2020). Its expression is strongly induced by chilling stress and the corresponding enzyme, while accepting a large range of substrates, has a strong affinity for nerolidol. Nerolidol glucoside has a higher ROS scavenging activity (DPPH assay) than its aglycone and possibly prevents lipid peroxidation to protect PSII during cold stress (Zhao et al., 2020). Glycosylation of volatile terpenoids allows their accumulation in non-volatile forms as a result of their higher water solubility, easing their storage in vacuoles (Yazaki et al., 2017). It is therefore possible that nerolidol glycosylation supports the biosynthesis of its aglycone form and finally contributes to plant cold tolerance.

While its substrate is so far unknown, UGT85A5 protects Arabidopsis plantlets from salt stress. Lines with ectopic expression of this gene indeed show lower loss of chlorophyll as compared to the wild-type (*ca*. 10% at 100 mM NaCl), concomitant with lower concentration of malondialdehyde (*ca*. + 40% in the wild-type at 300 mM NaCl), suggesting that the cells are more successful in keeping ROS at an acceptable level (Sun et al., 2013). *UGT85A5* is induced by salt, further suggesting that this gene is part of the plant response during salt exposure, as well as by abscisic acid (Rehman et al., 2018).

Results gathered from experiments investigating response and tolerance to abiotic stresses demonstrate the importance of several UGTs in this context. Those genes are generally induced under these conditions to mitigate the effects of oxidative stress. While this mechanism is now well described for flavonoids, such as quercetin and anthocyanins, it appears that other families, such as terpenoids (as illustrated here with nerolidol), may also partake in plant redox homeostasis. These findings prompt to study whether glycosylation of other families of molecules may be involved in plant response to unfavorable growth conditions. UGTs, by modulating the cellular repartition of targeted molecules, regulate their corresponding biosynthetic pathways, opening avenues for applications in managing abiotic stress tolerance.

## Phytohormones Glycosylation and Redox Status During Oxidative Stresses

Phytohormones undergo various conjugation reactions, with glycosylation being reported for substrates deriving from abscisic acid (ABA), gibberellins, strigolactones, cytokinins, auxins, brassinosteroids, salicylic acid, and jasmonic acid (Piotrowska and Bajguz, 2011; Islam et al., 2013; Maruri-López et al., 2019). These signaling molecules are key regulators with respect to plant acclimation to challenging environmental conditions. This section depicts the importance of phytohormone glycosylation in this context by using ABA, auxins and strigolactones as case studies.

### Abscisic Acid

ABA is one of the most thoroughly investigated molecules in the context of abiotic stresses, especially in response to drought (Takahashi et al., 2018; Gómez et al., 2019). Oxidative stress is a notable dimension explaining the cellular damages provoked by drought, especially through production of ^1^O_2_ by the photosynthetic electron transport chain in the PSII, resulting in oxidative damages in the chloroplasts (Noctor et al., 2014). The next paragraphs will therefore quickly emphasize the role of ABA in maintaining redox homeostasis during abiotic stresses, before to review the role of ABA glycosylation in terms of acclimation and oxidative damages.

Global transcriptome analyses of Arabidopsis after ABA application demonstrate the crucial role of this phytohormone during response to stress. Indeed, ontology analysis performed with Panther 15.0 (Mi et al., 2019) on Col-0 seedlings subjected to 10 µM ABA (**Supplementary Table S1A**) revealed the up-regulation of genes related to responses to water deprivation, osmotic stress, salt stress, cold, oxidative stress and stress (Goda et al., 2008). These results largely overlap those obtained by Hoth and colleagues (2002) on ecotype Landsberg plantlets cultivated in medium supplemented with 50 µM ABA (**Supplementary Table S1B**), where the biological processes responses to water deprivation, osmotic stress, salt stress, cold and stress were retrieved. ABA induces the expression of gene coding for transcription factors associated to tolerance to abiotic stress, such as for instance, *ZmHDZ10*, coding for a maize homeodomain–leucine zipper I (Zhao et al., 2014).

ABA-glucose ester (ABA-GE)/ABA equilibrium is considered as an important gatekeeper of plant response to abiotic stresses (Lee et al., 2006; Xu et al., 2012; Chen et al., 2020; Han et al., 2020). ABA-GE is an inactive form of ABA, together with hydroxylated conjugates, but this glycosylation is reversible (Nambara and Marion-Poll, 2005). ABA-GE accumulates in the vacuole and is therefore not able to activate the ABA signaling pathway. This glycosylation is caused by several abiotic stresses. For instance, the expression of an *UGT* from adzuki bean glycosylating ABA is significantly induced by dehydration in hypocotyls (Nambara and Marion-Poll, 2005). Furthermore, UGT71B6, UGT71B7, and UGT71B8 yield ABA-GE and their coding genes are induced by ABA, salt and osmotic stress (Lim et al., 2005; Dong et al., 2014). Their simultaneous silencing through RNAi results in plantlets more tolerant to osmotic stress and to lower water loss in excised plants, but also in smaller rosette leaves, shorter roots and pale green leaves in absence of stress (Dong et al., 2014). Hence, while silencing these *UGT*s responsible for ABA glycosylation results in higher tolerance to dehydration stress, it also penalizes plant growth under optimal conditions. We may therefore suggest that the biological functions of these UGT71s is to maintain an optimal ABA content under non-limiting growing conditions. Since they are ABA-, salt-, and osmotic-inducible, they may also mitigate ABA response under these conditions. A functional analysis of a gene closely related to *UGT71B6*/*7*/*8*, *UGT71C5*, reveals similar mechanisms of drought tolerance (Liu et al., 2015). Plants overexpressing *UGT71C5* are less drought-tolerant, with the *ugt71c5* mutant and downregulated lines showing the opposite phenotype. This lower expression of *UGT71C5* results in increased expression of ABA-responsive genes. In addition, *ugt71c5* mutant displays higher drought resistance than *ugt71b6* mutant (Liu et al., 2015). Finally, UGT75B1 is also able to yield ABA-GE, and overexpression of its coding gene results in impaired response to salt and drought stress, downregulation of several ABA-regulated genes involved in stress response and lower ABA content in detached rosette leaves (Chen et al., 2020). ABA-GE may play a key role in desiccation tolerance, since the β-D-glucosidase activity yielding ABA from ABA-GE is enhanced by salinity in several species (Nambara and Marion-Poll, 2005).

By contrast with *de novo* ABA biosynthesis, production of ABA from ABA-GE requires a single enzymatic reaction, which is catalyzed by several β-glucosidases in Arabidopsis. This simple hydrolysis step allows fast plant response when facing adverse challenging environmental conditions. For instance, mutants impaired in *BGLU33*, coding for a β-glucosidase active on ABA-GE, are more sensitive to drought and salt stresses than wild-type, while overexpressing lines are more tolerant (Xu et al., 2012). Similarly, mutant deficient in *BGLU18* is dwarf and highly sensitive to drought stress (Lee et al., 2006). Drought stress enhances BGLU18 activity through homomeric interaction, resulting in a 10-mer complex. This conformation drastically increases ABA content under drought stress as a result of ABA-GE hydrolysis (Lee et al., 2006). Recently, it was shown that the BGLU18 subcellular distribution is modified upon stress conditions (Han et al., 2020). In normal conditions, BGLU18 is mainly found in ER bodies. Following dehydration, osmotic or salt stress, the number of ER bodies significantly increases. These structures consist in temporary storage compartment that release BGLU upon stress, resulting in increased BGLU18-mediated ABA-GE hydrolysis. In addition, there is a relative increase in the BGLU18-microsomal fraction under stress, which also triggers ABA-GE hydrolysis activity. Upon stress, the following mechanisms explain altogether the increased ABA content resulting from BGLU18 activity: i) BGLU18 is organized in an active 10-mer complex, ii) BGLU18 is released from ER bodies, and iii) ABA-GE stored in the vacuole or in apoplastic space is transported to close vicinity of BGLU18.

Altogether, these results highlight the importance of ABA homeostasis and glycosylation status in tolerance to water deprivation or salt stress and subsequent mitigation of oxidative injuries.

### Auxins

Besides their developmental roles, auxins translate environmental and stress inputs into adequate plant response (Ludwig-Müller, 2011; Casanova-Sáez and Voß, 2019). The following paragraphs aim at explaining how auxin glycosylation may shape plant response to oxidative stress rising from abiotic constrains.

UGT74E2 enhances, among other biological processes, tolerance to drought and salt stress through glycosylation of the auxin indole-3-butyric acid (IBA), as demonstrated with recombinant enzymatic activity (Tognetti et al., 2010). The importance of UGT74E2 regarding oxidative stress is highlighted in one of the most relevant genetic backgrounds in relation to redox homeostasis, the Arabidopsis mutant deficient in CATALASE 2 (*cat2*), which is a crucial protein for H_2_O_2_ catabolism in peroxisomes (Pan et al., 2020). When grown in conditions favoring photorespiration and concomitant H_2_O_2_ accumulation, such as high light, 185 genes which were hardly expressed in conditions repressing photorespiration undergo a massive up-regulation (fold change > 21) in *cat2* (He et al., 2018). Supporting an important role for UGTs in response to H_2_O_2_ accumulation, five of them were part of these strongly induced genes, including *UGT73B4*, *UGT73B5*, *UGT73C4*, *UGT74E1*, and *UGT74E2*. *UGT74E2* is upregulated by osmotic, oxidative, ultraviolet B and salt stress (Tognetti et al., 2010). Its overexpression increases not only IBA-Glc content, but also free IBA, and modified the levels of IAA-Glc, IAA-Glu, and oxIAA, resulting in a shoot branching phenotype because of loss of apical dominance, often related to altered auxin homeostasis, as well as improved survival under drought and salt stresses (Tognetti et al., 2010). Collectively, these results suggest that UGT74E2 modifies auxin gradient and distribution to support plant acclimation under drought and salt stress conditions (Tognetti et al., 2010). This mechanism may rely on mutual interactions between flavonoids (especially flavonols) and auxin (Brunetti et al., 2018). More specifically, auxin induces flavonols biosynthesis, which in turn regulate auxin movement and signaling at the cellular and tissue levels by inhibiting the activity of several auxin transport proteins (such as PIN1, PIN2, and PIN5) and modifying the activity of auxin inactivation proteins (e.g., DIOXYGENASE FOR AUXIN OXIDATION). This circuit then invokes a panel of reactions in organelles facing oxidative stress, including chloroplasts, peroxisomes and nucleus (Brunetti et al., 2018). Auxin involvement in redox homeostasis remains however a highly complex picture with several parallel mechanisms whose relative importance remains to be investigated and hierarchized.

UGT85U1 has been isolated from *Crocus sativus* and is involved in response to abiotic stress (Ahrazem et al., 2015). The expression of this gene is inducible by salt, drought and cold stresses. Heterologous overexpression of *UGT85U1* in Arabidopsis resulted in plants with higher total chlorophyll content and increased resistance to oxidative stress caused by H_2_O_2_ exposure and salt stress as measured by growth parameters. LC-ESI/MS^n^ analysis revealed an increased content of free and conjugated forms of indole acetic acid (such as IAA-Glc and oxIAA) in the roots, suggesting that auxin may be an *in vivo* substrate of UGT85U1 (Ahrazem et al., 2015), although the authors did not demonstrate this finding with recombinant UGT85U1 assay.

By contrast with ABA, ontology analysis including genes significantly upregulated by auxin (**Supplementary Tables S1C, D**) highlights the absence of biological processes related to response to (a)biotic stresses, in an experiment directly treating Arabidopsis Col-0 seedlings with 1 µM IAA (Goda et al., 2008), and in roots from Arabidopsis Col-0 plantlets transferred to 1 µM IAA medium (Lewis et al., 2013). These transcriptomic analyses underline that auxin does not induce *per se* the expression of genes involved in redox homeostasis and response to stress. Instead, the relative proportion of conjugates and catabolites regulates auxin transport, signaling and plant response to various abiotic stresses.

### Strigolactones

Resurrection plants illustrate the ability of various species to adaptation to extreme environmental condition, particularly concerning drought stress. The functional characterization of an UGT isolated from the resurrection grass *Sporobolus stapfianus* illustrates how vital may be a glycosylation (Islam et al., 2013). This UGT is referred to as SDG8i and belongs to the UGT88 family. A protein extract from tobacco leaves infiltrated with an actin promoter-driven *SDG8i* expression is able to glycosylate the strigolactone analogue GR24, while very weak activity was retrieved in leaves infiltrated with the empty vector. No significant activities were detected for other phytohormones. *SDG8i* expression is drastically increased under severe water deficit. The heterologous overexpression of this gene in Arabidopsis results in higher tolerance to salt and drought stresses, as well as increased survival rate following a freezing period (at −8°C and −12°C) (Islam et al., 2013). It is hypothesized that strigolactones positively regulate plant response to drought and salt stress (Ha et al., 2014) and several elements suggest a strigolactones cross-talk with ABA sensitivity and biosynthesis (Cardinale et al., 2018). While the outreach of strigolactone glycosylation remains poorly understood, it was established that strigolactone strongly induces the expression of several key genes of the anthocyanin pathway, such as MYB transcription factors and downstream biosynthesis genes, raising anthocyanin content in Arabidopsis seedlings (Wang et al., 2020). Glycosylation may ease strigolactone translocation between distant organs and induce subsequent anthocyanin biosynthesis upon various abiotic stresses to protect specific tissues, such as leaves, from oxidative damages. SDG8i may therefore be involved in the regulation of strigolactone biosynthesis, which may be of crucial importance during extreme water deficit.

## Conclusion and Perspectives

Oxidative stress results from a wide range of environmental and physiological situations. It is therefore not surprising that plants have designed a large panel of response at many levels to address this deleterious cellular status. As underlined by the large molecule spectrum that may undergo glycosylation, especially phenolics and phytohormones, UGTs and glucosidases are able to support rapid and efficient plant response to numerous environmental conditions. The functional study of genes devoted to ROS detoxication such as CATs, SODs, and soluble peroxidases has demonstrated their importance to maintain redox homeostasis. Antioxidant molecules (such as flavonoids) are also widely acknowledged as efficient players in this respect, notably during abiotic stress events. Redox status is therefore a central hub connecting enzymatic ROS scavenging to biosynthesis and repartition of antioxidant/signaling molecules (partially regulated by glycosylation) in response to various endogenous and exogenous stimuli. The consequences of glycosylation regarding plant response to oxidative stress are multiple. This reaction may i) directly modify the antioxidant property of a molecule, such as flavonoids, ii) favor the biosynthesis of its corresponding aglycone by changing its subcellular location, such as scopolin and scopoletin, and iii) impact on phytohormones translocation, their signaling capacity and downstream gene expression regulation. It is also remarkable that the expression of many *UGTs* is stress-inducible and contributes to the plasticity of plants facing (a)biotic stresses, highlighting their potential valorization for designing plants more tolerant to sub-optimal growing conditions. Humankind benefits from antioxidant molecules, as their biochemical properties are not restricted to plants and strongly contribute to the nutritional values of various fruits and vegetables (Wang and Frei, 2011).

These findings have to be put in perspective with the considerable efforts which have been produced to decipher the molecular basis of redox signaling, now reported to play an important role during plant response to adverse environmental and/or developmental conditions and more specifically to oxidative stress (Waszczak et al., 2018; Farooq et al., 2019). The chloroplastic compartment, which is the main source of ROS formation under light conditions (Vaahtera et al., 2014), is a sensitive probe of cellular redox status. Under higher accumulation of oxidant markers, such as H_2_O_2_, chloroplast is able to trigger a signaling cascade leading to expression of nuclear genes to counterbalance the negative effects of oxidant conditions and rescue the functions of not only chloroplasts, but also mitochondria (Leister, 2019).

This ROS-dependent signaling modifies the nuclear transcriptome, but also initiates post-transcriptional, translational, and post-translational regulatory mechanisms (He et al., 2018). ROS may activate these pathways through changes of targeted proteins in either, their conformational state (monomerization, polymerization), interaction with a partner, cleavage from an organelle to allow translocation to the nucleus, enhancement of their DNA binding activity, phosphorylation through the mitogen-activated protein kinase cascade, but also through regulation of general transcription factor activity, such as Mediator (He et al., 2018).

Therefore, any modification related to redox homeostasis, such as concentration of oxidant and antioxidant molecules or proteins, may potentially result in repression or activation of retrograde signaling pathways. Ultimately, it is also possible that impairing the redox homeostasis mechanism causes better resistance to oxidative stress through activation of a retrograde signaling pathway. Since several UGTs are known to regulate the content of specific flavonoids (Chong et al., 2002; Zhao et al., 2019), for instance, in the chloroplasts (Agati et al., 2012), we may hypothesize that this modification of the non-enzymatic ROS scavenging capacity interacts with the chloroplast retrograde signaling wave. Such associations deserve further investigation, especially regarding adaptation required to face the increasingly challenging environmental conditions of the Anthropocene era (Basso et al., 2018).

## Author Contributions

MBe designed the review, conducted literature review, and wrote the manuscript. GN, MJ, and MBa reviewed and critically revised the manuscript. All authors contributed to the article and approved the submitted version.

## Funding

MBe is supported by Belgian Fonds de la Recherche Scientifique (FRS-FNRS) research project T.0068.18. MBa is a Senior Research Associate of the FRS-FNRS.

## Conflict of Interest

The authors declare that the research was conducted in the absence of any commercial or financial relationships that could be construed as a potential conflict of interest.
